# 1,2-Bis[(1,3-benzodioxol-5-yl)methyl­idene]hydrazine

**DOI:** 10.1107/S1600536811050793

**Published:** 2011-12-03

**Authors:** Jerry P. Jasinski, James A. Golen, A. S. Praveen, B. Narayana, H. S. Yathirajan

**Affiliations:** a Department of Chemistry, Keene State College, 229 Main Street, Keene, NH 03435-2001, USA; b Department of Studies in Chemistry, University of Mysore, Manasagangotri, Mysore 570 006, India; c Department of Studies in Chemistry, Mangalore University, Mangalagangotri, 574 199, India

## Abstract

The complete mol­ecule of the title compound, C_16_H_12_N_2_O_4_, is generated by the application of a centre of inversion. The (1,3-benzodioxol-5-yl)methyl­idene fused-ring system is approximately planar (r.m.s. deviation = 0.020 Å) and is essentially coplanar with the central hydrazine group [dihedral angle = 5.08 (9)°]. Weak π–π inter­molecular inter­actions are observed [centroid–centroid distance = 3.8553 (8) Å], providing some packing stability.

## Related literature

For the biological activity of Schiff bases, see: Aydogan *et al.* (2001 ▶); Desai *et al.* (2001 ▶); El-Masry *et al.* (2000 ▶); Hodnett & Dunn (1970 ▶); Pandey *et al.* (1999 ▶); Singh & Dash (1988 ▶); Taggi *et al.* (2002 ▶); Xu *et al.* (1997 ▶). For the crystallography and coordination chemistry of compounds containing the azine functionality or a diimine linkage, see: Xu *et al.* (1997 ▶); Kundu *et al.* (2005 ▶). For related structures, see: Liu *et al.* (2007 ▶); Odabaşoğlu *et al.* (2007 ▶); Zhang & Zheng (2008 ▶); Zheng *et al.* (2005*a* ▶,*b* ▶).
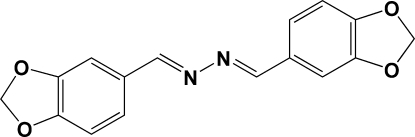


## Experimental

### 

#### Crystal data

C_16_H_12_N_2_O_4_
*M* *_r_* = 296.28
Monoclinic, 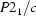

*a* = 6.1835 (2) Å
*b* = 4.5970 (2) Å
*c* = 23.8487 (10) Å
β = 96.080 (4)°
*V* = 674.10 (5) Å^3^
*Z* = 2
Mo *K*α radiation
μ = 0.11 mm^−1^
*T* = 170 K
0.28 × 0.25 × 0.08 mm

#### Data collection

Oxford Diffraction Xcalibur Eos Gemini diffractometer
Absorption correction: multi-scan (*CrysAlis RED*; Oxford Diffraction, 2010 ▶) *T*_min_ = 0.971, *T*_max_ = 0.992
4759 measured reflections
1746 independent reflections
1402 reflections with *I* > 2σ(*I*)
*R*_int_ = 0.019

#### Refinement

*R*[*F* ^2^ > 2σ(*F* ^2^)] = 0.043
*wR*(*F* ^2^) = 0.118
*S* = 1.04
1746 reflections
100 parameters
H-atom parameters constrained
Δρ_max_ = 0.20 e Å^−3^
Δρ_min_ = −0.18 e Å^−3^

### 

Data collection: *CrysAlis PRO* (Oxford Diffraction, 2010 ▶); cell refinement: *CrysAlis PRO*; data reduction: *CrysAlis RED* (Oxford Diffraction, 2010 ▶); program(s) used to solve structure: *SHELXS97* (Sheldrick, 2008 ▶); program(s) used to refine structure: *SHELXL97* (Sheldrick, 2008 ▶); molecular graphics: *SHELXTL* (Sheldrick, 2008 ▶); software used to prepare material for publication: *SHELXTL*.

## Supplementary Material

Crystal structure: contains datablock(s) global, I. DOI: 10.1107/S1600536811050793/tk5026sup1.cif

Structure factors: contains datablock(s) I. DOI: 10.1107/S1600536811050793/tk5026Isup2.hkl

Supplementary material file. DOI: 10.1107/S1600536811050793/tk5026Isup3.cml

Additional supplementary materials:  crystallographic information; 3D view; checkCIF report

## Figures and Tables

**Fig. 1.**
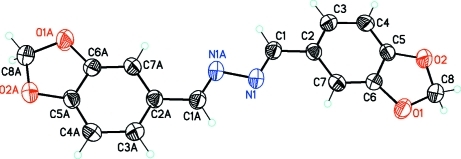
Molecular structure of the title compound showing the atom labeling scheme and 50% probability displacement ellipsoids.

**Fig. 2.**
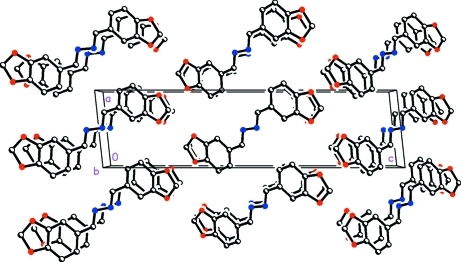
Packing diagram of the title compound viewed along the b axis. The H atoms have been removed for clarity.

## supplementary crystallographic information

### Comment

Schiff bases are used as substrates in the preparation of a number of industrial and biologically active compounds via ring closure, cycloaddition and replacement reactions. Moreover, Schiff bases are also known to have biological activities such as antimicrobial (El-Masry *et al.*, 2000 & Pandey *et al.*, 1999), antifungal (Singh & Dash, 1988), antitumor (Hodnett & Dunn, 1970; Desai *et al.*, 2001), and as herbicides. Schiff bases have also been employed as ligands for complexation of metal ions (Aydogan *et al.*, 2001). On the industrial scale, they have a wide range of applications such as dyes and pigments (Taggi *et al.*, 2002). Compounds containing an azine functionality or a diimine linkage have been investigated in terms of their crystallography and coordination chemistry (Xu *et al.*, 1997; Kundu *et al.*, 2005).

The crystal structures of some Schiff base hydrazines, viz., 4-fluorobenzaldehyde [(*E*)-4-fluorobenzylidene]hydrazone (Odabaşoğlu *et al.*, 2007), *N*,*N*'-bis(3 nitrobenzylidene)hydrazine (Zheng *et al.*, 2005*a*), *N*,*N*'-bis(4-chlorobenzylidene)hydrazine (Zheng *et al.*, 2005*b*), 1,2-bis(2-chlorobenzylidene)hydrazine (Zhang & Zheng, 2008), *N*,*N*'-bis(4-hydroxybenzylidene)hydrazine (Liu *et al.*, 2007) have been reported. In view of the importance of Schiff base hydrazines, the crystal structure of title compound (I) is reported

In the crystal structure of the title compound, C_16_H_12_N_2_O_4_, the two 1,3-benzodioxol-5-ylmethylidene rings are planar to the hydrazine group and to each other (Fig. 1). Weak π–π intermolecular interactions are observed (centroid–centroid distance = 3.8553 (8) Å) providing some packing stability (Fig. 2).

### Experimental

A mixture of piperanal (3.0 g, 0.02 mol) and hydrazine hydrate (0.6 ml, 0.012 mol) was refluxed in 15 ml of absolute alcohol containing 2 drops of sulfuric acid, for about 3 hours. On cooling, the solid separated was filtered and dried. Single crystals were grown from DMF by slow evaporation. Yield: 81%. (*M*.pt.: 476 K).

### Refinement

All of the H atoms were placed in their calculated positions and then refined using the riding model with C—H lengths of 0.93 Å (CH) or 0.97 Å (CH_2_), and with *U*_iso_(H) set to 1.19-1.20 (CH, CH_2_) × *U*_eq_(parent atom).

### Crystal data

**Table:**

C_16_H_12_N_2_O_4_	*F*(000) = 308
*M**_r_* = 296.28	*D*_x_ = 1.460 Mg m^−^^3^
Monoclinic, *P*2_1_/*c*	Mo *K*α radiation, λ = 0.71073 Å
Hall symbol: -P 2ybc	Cell parameters from 1659 reflections
*a* = 6.1835 (2) Å	θ = 3.4–30.1°
*b* = 4.5970 (2) Å	µ = 0.11 mm^−^^1^
*c* = 23.8487 (10) Å	*T* = 170 K
β = 96.080 (4)°	Plate, yellow
*V* = 674.10 (5) Å^3^	0.28 × 0.25 × 0.08 mm
*Z* = 2	

### Data collection

**Table:**

Oxford Diffraction Xcalibur Eos Gemini diffractometer	1746 independent reflections
Radiation source: Enhance (Mo) X-ray Source	1402 reflections with *I* > 2σ(*I*)
graphite	*R*_int_ = 0.019
Detector resolution: 16.1500 pixels mm^-1^	θ_max_ = 30.1°, θ_min_ = 3.4°
ω scans	*h* = −8→8
Absorption correction: multi-scan (*CrysAlis RED*; Oxford Diffraction, 2010)	*k* = −5→6
*T*_min_ = 0.971, *T*_max_ = 0.992	*l* = −32→23
4759 measured reflections	

### Refinement

**Table:**

Refinement on *F*^2^	Primary atom site location: structure-invariant direct methods
Least-squares matrix: full	Secondary atom site location: difference Fourier map
*R*[*F*^2^ > 2σ(*F*^2^)] = 0.043	Hydrogen site location: inferred from neighbouring sites
*wR*(*F*^2^) = 0.118	H-atom parameters constrained
*S* = 1.04	*w* = 1/[σ^2^(*F*_o_^2^) + (0.0592*P*)^2^ + 0.1301*P*] where *P* = (*F*_o_^2^ + 2*F*_c_^2^)/3
1746 reflections	(Δ/σ)_max_ < 0.001
100 parameters	Δρ_max_ = 0.20 e Å^−^^3^
0 restraints	Δρ_min_ = −0.18 e Å^−^^3^

### Special details

**Table:**

Geometry. All esds (except the esd in the dihedral angle between two l.s. planes) are estimated using the full covariance matrix. The cell esds are taken into account individually in the estimation of esds in distances, angles and torsion angles; correlations between esds in cell parameters are only used when they are defined by crystal symmetry. An approximate (isotropic) treatment of cell esds is used for estimating esds involving l.s. planes.
Refinement. Refinement of *F*^2^ against ALL reflections. The weighted *R*-factor *wR* and goodness of fit *S* are based on *F*^2^, conventional *R*-factors *R* are based on *F*, with *F* set to zero for negative *F*^2^. The threshold expression of *F*^2^ > σ(*F*^2^) is used only for calculating *R*-factors(gt) *etc*. and is not relevant to the choice of reflections for refinement. *R*-factors based on *F*^2^ are statistically about twice as large as those based on *F*, and *R*- factors based on ALL data will be even larger.

### Fractional atomic coordinates and isotropic or equivalent isotropic displacement parameters (Å^2^)

**Table:**

	*x*	*y*	*z*	*U*_iso_*/*U*_eq_	
O1	0.40433 (16)	0.1982 (3)	0.30379 (4)	0.0593 (4)	
O2	0.05345 (15)	0.0196 (2)	0.29688 (4)	0.0480 (3)	
N1	0.48668 (18)	0.9022 (3)	0.47706 (4)	0.0426 (3)	
C1	0.2915 (2)	0.8116 (3)	0.46809 (5)	0.0382 (3)	
H1A	0.1891	0.8790	0.4909	0.046*	
C2	0.22381 (19)	0.6056 (3)	0.42323 (5)	0.0347 (3)	
C3	0.0129 (2)	0.4965 (3)	0.41852 (5)	0.0400 (3)	
H3A	−0.0823	0.5609	0.4435	0.048*	
C4	−0.0601 (2)	0.2930 (3)	0.37729 (6)	0.0417 (3)	
H4A	−0.2004	0.2178	0.3747	0.050*	
C5	0.0850 (2)	0.2105 (3)	0.34108 (5)	0.0358 (3)	
C6	0.2948 (2)	0.3188 (3)	0.34523 (5)	0.0367 (3)	
C7	0.36960 (19)	0.5151 (3)	0.38540 (5)	0.0385 (3)	
H7A	0.5111	0.5862	0.3877	0.046*	
C8	0.2520 (2)	0.0174 (4)	0.27125 (6)	0.0469 (4)	
H8A	0.3079	−0.1794	0.2701	0.056*	
H8B	0.2271	0.0900	0.2329	0.056*	

### Atomic displacement parameters (Å^2^)

**Table:**

	*U*^11^	*U*^22^	*U*^33^	*U*^12^	*U*^13^	*U*^23^
O1	0.0448 (6)	0.0813 (9)	0.0538 (6)	−0.0115 (5)	0.0141 (5)	−0.0309 (6)
O2	0.0443 (5)	0.0526 (7)	0.0463 (6)	−0.0050 (4)	0.0008 (4)	−0.0174 (5)
N1	0.0472 (6)	0.0415 (7)	0.0384 (6)	−0.0023 (5)	0.0016 (5)	−0.0110 (5)
C1	0.0428 (7)	0.0355 (7)	0.0360 (6)	0.0014 (5)	0.0027 (5)	−0.0033 (5)
C2	0.0375 (6)	0.0323 (7)	0.0335 (6)	0.0014 (5)	−0.0005 (5)	−0.0003 (5)
C3	0.0372 (6)	0.0429 (8)	0.0406 (7)	0.0009 (5)	0.0067 (5)	−0.0035 (6)
C4	0.0321 (6)	0.0450 (8)	0.0476 (7)	−0.0035 (5)	0.0023 (5)	−0.0043 (6)
C5	0.0370 (6)	0.0340 (7)	0.0347 (6)	0.0006 (5)	−0.0036 (5)	−0.0020 (5)
C6	0.0356 (6)	0.0405 (7)	0.0342 (6)	0.0014 (5)	0.0039 (5)	−0.0020 (5)
C7	0.0327 (6)	0.0421 (8)	0.0402 (7)	−0.0036 (5)	0.0015 (5)	−0.0031 (5)
C8	0.0476 (8)	0.0507 (9)	0.0420 (7)	0.0016 (6)	0.0026 (6)	−0.0110 (6)

### Geometric parameters (Å, °)

**Table:**

O1—C6	1.3728 (15)	C3—C4	1.3970 (19)
O1—C8	1.4228 (17)	C3—H3A	0.9300
O2—C5	1.3697 (15)	C4—C5	1.3630 (18)
O2—C8	1.4284 (18)	C4—H4A	0.9300
N1—C1	1.2732 (17)	C5—C6	1.3836 (18)
N1—N1^i^	1.413 (2)	C6—C7	1.3607 (18)
C1—C2	1.4566 (18)	C7—H7A	0.9300
C1—H1A	0.9300	C8—H8A	0.9700
C2—C3	1.3907 (18)	C8—H8B	0.9700
C2—C7	1.4043 (18)		
			
C6—O1—C8	106.27 (10)	C4—C5—O2	128.05 (12)
C5—O2—C8	105.97 (10)	C4—C5—C6	121.98 (12)
C1—N1—N1^i^	111.66 (14)	O2—C5—C6	109.97 (11)
N1—C1—C2	121.97 (12)	C7—C6—O1	128.17 (12)
N1—C1—H1A	119.0	C7—C6—C5	122.35 (12)
C2—C1—H1A	119.0	O1—C6—C5	109.48 (11)
C3—C2—C7	120.11 (12)	C6—C7—C2	117.09 (11)
C3—C2—C1	119.19 (12)	C6—C7—H7A	121.5
C7—C2—C1	120.69 (12)	C2—C7—H7A	121.5
C2—C3—C4	121.90 (12)	O1—C8—O2	108.17 (11)
C2—C3—H3A	119.1	O1—C8—H8A	110.1
C4—C3—H3A	119.1	O2—C8—H8A	110.1
C5—C4—C3	116.56 (12)	O1—C8—H8B	110.1
C5—C4—H4A	121.7	O2—C8—H8B	110.1
C3—C4—H4A	121.7	H8A—C8—H8B	108.4
			
N1^i^—N1—C1—C2	−179.47 (14)	C8—O1—C6—C5	2.58 (16)
N1—C1—C2—C3	174.71 (13)	C4—C5—C6—C7	−0.3 (2)
N1—C1—C2—C7	−4.6 (2)	O2—C5—C6—C7	179.55 (12)
C7—C2—C3—C4	1.0 (2)	C4—C5—C6—O1	179.77 (13)
C1—C2—C3—C4	−178.40 (13)	O2—C5—C6—O1	−0.38 (16)
C2—C3—C4—C5	−1.2 (2)	O1—C6—C7—C2	179.93 (13)
C3—C4—C5—O2	−178.96 (13)	C5—C6—C7—C2	0.0 (2)
C3—C4—C5—C6	0.9 (2)	C3—C2—C7—C6	−0.3 (2)
C8—O2—C5—C4	177.86 (14)	C1—C2—C7—C6	179.01 (12)
C8—O2—C5—C6	−1.98 (15)	C6—O1—C8—O2	−3.78 (16)
C8—O1—C6—C7	−177.34 (15)	C5—O2—C8—O1	3.55 (16)

Symmetry codes: (i) −*x*+1, −*y*+2, −*z*+1.
